# Identification of a Glypican-3 Binding Peptide From a Phage-Displayed Peptide Library for PET Imaging of Hepatocellular Carcinoma

**DOI:** 10.3389/fonc.2021.679336

**Published:** 2021-06-04

**Authors:** Jiayao Yan, Xiaoxiao Yu, Xiaotong Chen, Fangcen Liu, Fangjun Chen, Naiqing Ding, Lixia Yu, Fanyan Meng, Jie Shen, Jia Wei, Baorui Liu

**Affiliations:** ^1^ The Comprehensive Cancer Centre of Nanjing Drum Tower Hospital, The Affiliated Hospital of Nanjing University Medical School, Nanjing, China; ^2^ The Comprehensive Cancer Centre of Nanjing Drum Tower Hospital, Clinical College of Nanjing Medical University, Nanjing, China; ^3^ The Comprehensive Cancer Centre, China Pharmaceutical University Nanjing Drum Tower Hospital, Nanjing, China; ^4^ The Department of Pathology, Nanjing Drum Tower Hospital, The Affiliated Hospital of Nanjing University Medical School, Nanjing, China

**Keywords:** phage display, tumor-targeting peptide, glypican-3, hepatocellular carcinoma, PET imaging

## Abstract

Tumor-targeting peptides functioned as molecular probes are essential for multi-modality imaging and molecular-targeting therapy in caner theronostics. Here, we performed a phage-displayed bio-panning to identify a specific binding peptide targeting Glypican-3 (GPC-3), a promising biomarker in hepatocellular carcinoma (HCC). After screening in the cyclic peptide library, a candidate peptide named F3, was isolated and showed specific binding to HCC cell lines. In a bio-distribution study, higher accumulation of F3 peptide was observed in HepG-2 tumors compared to PC-3 tumors in xenograft models. After labeling with radioactive ^68^Ga, the F3 peptide tracer enabled the specific detection of tumors in HCC tumor models with PET imaging. More importantly, the expression of GPC-3 in human tissue samples may be distinguished by an F3 fluorescent peptide probe indicating its potential for clinical application. This cyclic peptide targeting GPC-3 has been validated, and may be an alternative to serve as an imaging probe or a targeting domain in the drug conjugate.

## Introduction

Hepatocellular carcinoma (HCC) derived from malignant hepatocytes is among the most common cancers in adults. World-widely, the mortal cases of HCC account for 8.2% of all types of cancer, which is second to lung cancer, colorectal cancer and gastric cancer (1). Recently, some therapeutic approaches have been explored for HCC treatment, such as liver transplantation, molecular-targeting drugs and immunotherapy. Although these treatments are effective for a portion of patients, the survival rate of HCC is still unfavorable and the majority of patients are diagnosed at late stage of the first visit, suggesting poor prognosis (2–4). For this reason, accurate and precise approaches to early diagnosis of HCC are urgently needed.

Normally, the early diagnosis of HCC depends on liver biopsy, imaging examination and serum tests, including alpha-fetoprotein (AFP) in combination with other biomarkers (5, 6). Glypican-3 (GPC-3) is one of the heparin sulfate proteoglycans with a molecular weight of 70 kDa, that has been characterized as a sensitive and specific biomarker of HCC. It has been recognized that GPC-3 can be involved in cell proliferation, adhesion and migration through Wnt/β-Catenin, Hedgehog, YAP and other signaling pathways in HCC. The GPC-3 protein expressed on the cell membrane consists of two subunits connected by two heparin sulphate side chains after furin-cleavage (7). As an oncofetal protein, GPC-3 is primarily present during embryonic period, but absent in normal adult tissues, whereas it’s usually up-regulated in malignant tissues such as HCC (over 80%) (8) and AFP-producing gastric cancer (AFP-GC) (over 90%) (9). In addition, the expression of GPC-3 protein is often absent in cholangiocarcinoma (CCA) (10). Thus, its excellent tumor specificity and cell-membrane localization make GPC-3 a promising biomarker for precise diagnosis and targeting therapy of HCC.

To obtain a high affinity and specific binder to GPC-3, the tumor-targeting peptide with the advantage of synthesis and modification is an alternative to other than traditional antibodies (11, 12). Recently, phage-displayed libraries containing numerous randomized peptides on the surface of phages are becoming more popular for screening a specific binder to the selected target. For example, iRGD, Lyp-1, TT1, PL1 and other tumor-targeting peptides are generated from the phage display technology for directing delivery of anti-tumor payloads (13–16).

In this study, we used a cyclic phage-displayed peptide library with 9 amino acids considering that the annular conformation of peptide might contribute to the affinity between binder and target (17). Through screening the phage-displayed peptide library against a recombinant human GPC-3 protein, we identified a targeting peptide F3 which recognized GPC-3 both *in vitro* and *in vivo*, showing great potential as a functional probe in HCC PET imaging and pathological examination.

## Materials and Methods

### Ethics

This study was performed in accordance with the Declaration of Helsinki. The work obtained ethics approval by Nanjing Drum Tower Hospital Ethics Committee. All patients included were consented to participate in the study and to use their tissue samples in research. The ethics approval statements for animal work were provided by the Institutional Animal Care and Use Committee of Nanjing Drum Tower Hospital. The procedures for animal experiments were carried out in accordance with the Guide of Care and Use of Laboratory Animals, 8th Edition (2011).

### Cell Lines and Mice

The human hepatocellular carcinoma cell line HepG-2 (ATCC, Manassas, VA, USA, RRID: CVCL_0027) was cultured in DMEM medium (Thermo Scientific, Waltham, MA, USA) supplemented with 10% fetal bovine serum (Gibco, Grand Island, NY, USA) at 37°C in an atmosphere containing 5% CO_2_. The human prostate cancer cell line PC-3 (ATCC, Manassas, VA, USA, RRID: CVCL_0035) was cultured in DMEM/F-12 medium (Thermo Scientific, Waltham, MA, USA) supplemented with 10% fetal bovine serum (Gibco, Grand Island, NY, USA) at 37°C with 5% CO_2_.

In this study, all animal experiments were carried out according to the Institutional Animal Care and Use Committee (IACUC) guidelines and all studies followed the approved protocols by the IACUC at Nanjing Drum Tower Hospital. The male BALB/c nude mice (4–6 weeks old, male, 19–21 g) were purchased from Nanjing QingLongShan Laboratory Animal Technology Co., Ltd (Nanjing, China). Mice have free access to sterilized water and food, fed under a specific pathogen-free condition with controlled temperature (~25°C), humidity (50–70%) and circadian rhythm (a 12-h light/dark cycle). All essential procedures were performed to minimize discomfort and avoid waste of animals. HepG-2 and PC-3 tumor models were generated by subcutaneous injection of 7 × 10^6^ cells into the front flank of male athymic nude mice. The length and width were measured every other day and tumor volume was calculated following the formula: volume = length × width × width × 0.5. The mice were subjected to bio-distribution studying or positron emission tomography imaging after the tumor volume reached 150 to 200 mm^3^.

### *In Vitro* Phage Display

The Ph.D.-C7C phage display library was bio-panned following the manufacturer’s protocol (New England Biolabs, Ipswich, MA, USA) with moderate modification. The library displayed seven random peptides between two cysteine to form a cyclic loop and was ligated at the N-terminus of the minor coat protein (pIII) of M13 phage. Briefly, the recombinant human GPC-3 protein (Sino Biological, Beijing, China) as a target for bio-panning was immobilized on a polystyrene dish at a concentration of 100 μg/ml and shook gently at a temperature of 4°C overnight. Subsequently, the target protein immobilized on the dish was blocked by 0.5% (w/v) BSA at 37°C for 1 h. After being blocked, the dish was quickly washed with PBST containing 0.1% (v/v) Tween-20 for six times. And then, 10 μl primary phage-displayed peptide library (2 × 10^13^ pfu per ml) dissolved in 2 ml 0.1% PBST as input phages were incubated with GPC-3 protein for 2.5 h at room temperature. After incubation, unbound phages were washed off with 0.1% PBST for 10 times, and bound phages as output phages were eluted with 0.2M Glycine-HCl (pH = 2.2) containing 1 mg/ml BSA at 4°C for 20 min. After neutralized with 1 M Tris-HCl (pH = 9.1), 20 μl of eluted phages was collected and tittered by a blue plaque-forming assay on LB/IPTG/Xgal plates. The remaining phages were amplified and amplification was also tittered to decide the number of input phages for next round bio-panning. The enrichment rate was calculated from the output/input ratio of phages recovered after each round of bio-panning. This bio-panning procedure was performed repeatedly three more times. During four rounds of bio-panning, the concentration of Tween-20 in washing buffer was increased stepwise (0.1%–0.75%) to ensure reinforcement of affinity. Finally, foreign DNA regions of the bio-panned phages were sequenced using -96 gIII sequencing primer (5ˊ-HOCCC TCA TAG TTA GCG TAA CG-3ˊ), and corresponding sequences of phage-displayed peptides were analyzed based on a DNA map provided by manufacturer’s instruction.

## Enzyme-Linked Immunosorbent Assay

The selected phages were amplified and purified for enzyme linked immunosorbent assay (ELISA). In protein ELISA, the recombinant human GPC-3 protein (10 μg/well) as the target protein was immobilized on a 96-well plate at 4°C overnight. Next day, the selected phages of eight gradients diluted titers (four fold between neighboring grades) were added into each well to incubate with GPC-3 protein at room temperature for 3 h (n = 3/titer). After washing steps, the anti-M13-horseradish peroxidase (anti-M13-HRP) (Sino Biological, Beijing, China, RRID: AB_10764206) was added and mixed with the selected phages. After the reaction between anti-M13-HRP and M13 phage at room temperature for 1 h, 100 μl 3,3ˊ,5,5ˊ-tetramethylbenzidine (TMB) (TransGen, Beijing, China, RRID: AB_2336758) was added to each well and kept in the dark for 2 min at room temperature. To stop the reaction, 100 μl of 1 M hydrochloric acid was added into each well, and then, the absorbance at 450 nm was measured by an automatic microplate reader (Tecan, Männedorf, Switzerland). In the cell ELISA, HepG-2 and PC-3 cells as targets were respectively seeded in a 96-well plate at a concentration of 2 × 10^5^ cells/well. The subsequent procedures followed the protein ELISA described above. The affinity measurement was also performed following the ELISA procedures (15).

## Fluorescent Cell Staining

To assess the binding ability of peptide, F3 cyclic peptide and C-G7-C cyclic peptide (sequence: CGGGGGGGC) were synthesized and labeled with Rhodamine B (RhoB) (ChinaPeptides, Shanghai, China). Briefly, peptides were synthesized with an automatic microwave assisted peptide synthesizer (ChinaPeptides, Shanghai, China) using standard solid-phase Fmoc/t-Bu chemistry and labeled with RhoB consequently. For cell immunofluorescence staining, HepG-2 and PC-3 cells were respectively seeded into a 12-well plate at the concentration of 1.5 × 10^4^ cells/well. Next day, cells were fixed with 4% paraformaldehyde (PFA) for 25 min at room temperature. And then, the cells were blocked by 1% (w/v) BSA at 37°C for 1 h. To detect the expression of GPC-3 proteins, HepG-2 and PC3 cells were incubated with mouse anti-GPC-3 primary antibody (Abcam, Cambridge, MA, USA, RRID: AB_2476152) for 2 h at 37°C. After washing with PBS, HepG-2 and PC-3 cells were visualized with FITC-conjugated goat anti-mouse secondary antibody (Beyotime, Shanghai, China, RRID: AB_11187492). In a cell binding assay, HepG-2 and PC-3 cells were incubated with 10 μM RhoB-peptide at 37°C for 2 h after being fixed and blocked as described above. To further confirm the specific binding between F3 peptide and GPC-3 protein, the fixed HepG2 cells were blocked by 1% BSA containing anti-GPC3 primary antibody (Abcam, Cambridge, MA, USA, RRID: AB_2476152) for 1 h prior to RhoB-F3 incubation as a cell blocking assay. After labeling the nuclei with 4’, 6-diamidino-2-phenylindole (DAPI, Beyotime, Shanghai, China), the cells were observed with an EVOS fluorescence microscope (Thermo Scientific, Waltham, MA, USA). The semi-quantitative analysis of fluorescence presented as integrated optical density (IOD) was analyzed by Image J software (National Institutes of Health, Maryland, USA).

### Bio-Distribution of F3 Peptide

Nude mice bearing HepG-2 or PC-3 tumor were established as described above. RhoB labeled F3 peptide was intravenously injected through tail vein and allowed to circulate for 1 h before scarification. After anesthesia, tumors and several major organs were harvested and prepared for sections. Subsequently, cryosections were made out of frozen tissues. After staining with DAPI for nuclei, fluorescence of sections were detected with an EVOS fluorescence microscope (Thermo Scientific, Waltham, MA, USA). The harvested tissues were also fixed in 4% PFA and sectioned for HE staining. HE images of each slice were obtained under a ZEISS microscope (ZEISS, Baden-Württemberg, Germany). The images of each slice were analyzed by Image J software (National Institutes of Health, Maryland, USA) as described above.

### Construction of Radioactive Tracer Labeled With ^68^Ga

For labeling with radioactive ^68^Ga, F3 peptide modified with 1,4,7,10-tetraazacyclododecane-1,4,7,10-tetraacetic acid (DOTA) was synthesized (Genscript, Nanjing, China). The ^68^Ga-DOTA-F3 probe was constructed according to the synthetic scheme shown in **Figure 5A**. Briefly, DOTA-peptide was dissolved in 0.25 M sodium acetate buffer, and then mixed with the ^68^GaCl_3_ eluted from a ^68^Ge-^68^Ga radionuclide generator (ITG, Germany) with 0.05 M HCl. The mixture was incubated at 95°C for 10 min and cooled gradually. The ^68^Ga-DOTA-F3 product was purified through a C18 plus light cartridge (Waters, Sep-Pak, Milford, USA) while free ^68^Ga was collected and dropped into waste tube. The product attached to C18 plus cartridge was eluted with 60% ethanol. After washing with saline solution, the ^68^Ga-DOTA-F3 product was collected in product tube. For purity evaluation, the radioactivity of the waste tube, C18 plus cartridge and product tube was individually measured by a CRC-15R radioactivity detector (CAPINTEC, New Jersey, USA). The labeling efficiency (Radio-chemical yield, RCY) was calculated following the formula: Radio-chemical yield (%) = Product radioactivity/Input radioactivity.

### Positron Emission Tomography Imaging

HepG-2 xenograft mice were established as described above. The mice were intravenously injected with 7.40 MBq of ^68^Ga-DOTA-F3 tracer through the tail vein. During circulation, positron emission tomography (PET) imaging was acquired at different time points (30, 60, and 120 min after circulation). In a blocking assay, 100 µg of the unlabeled F3 peptide was intravenously injected at 60 min prior to the radioactive tracer injection. All PET scans were performed on the Inveon micro-PET/CT scanner (Siemens, Berlin, Germany). All PET images were analyzed by the Inveon Research Workplace (Siemens, Berlin, Germany). Region of interest (ROI) of each image was drawn using a three dimensional (3D) volume mode. All ROIs data was automatically normalized to %ID/g by Inveon Research Workplace.

### GPC-3 Detection in Human Tissue Samples

All human tissue samples applied in this study were obtained from the Department of Pathology in Nanjing Drum Tower Hospital. This study was performed in accordance with the Declaration of Helsinki. All patients included were consented to participate in the study and to use their tissue samples in research. Our procedures were approved by the Ethics Committee of Nanjing Drum Tower Hospital. To detect the expression of GPC-3 protein in human tissue samples, tumor tissues from HCC, CCA, AFP-GC patients and several normal adult organ tissues were collected and diagnosed by pathologists. To evaluate the expression of GPC-3 in different cancer, all cancer tissues were immunohistochemically (IHC) stained with anti-GPC-3 antibody (Abcam, Cambridge, MA, USA, RRID: AB_2476152) and visualized by an ABC peroxidase standard staining kit (Thermo Scientific, Waltham, MA, USA). Expression levels of GPC-3 were evaluated according to the criterion of pathology. To assess the binding capacity of RhoB-F3, fluorescence staining was performed on the above tissue samples. Rho-G7 was also applied in staining as a negative control. Tissue slices were blocked by 5% BSA at room temperature for 1 h. After washing steps, tissue slices were incubated with 100 μM RhoB-peptide at room temperature for 2 h. To remove unbound fluorescent peptide, slices were washed by 0.5% (v/v) Tween-20 in PBS for five times. After mounting with DAPI, all slices were observed with an EVOS FL Auto fluorescence microscope (Thermo Scientific, Waltham, MA, USA). The images of each slice were analyzed by Image J software (National Institutes of Health, Maryland, USA) as described above.

### Statistical Analysis

All data analysis in this study was performed on Graphpad Prism 6.0 (Graphpad software, San Diego, CA). The Student’s t test was used for analysis of each paired experiment. Data were presented as mean ± SD unless indicated specifically. P < 0.05 was considered statistically significant.

## Results

### Identification of the GPC-3 *S*pecifically Binding Clones

**Figure 1:**
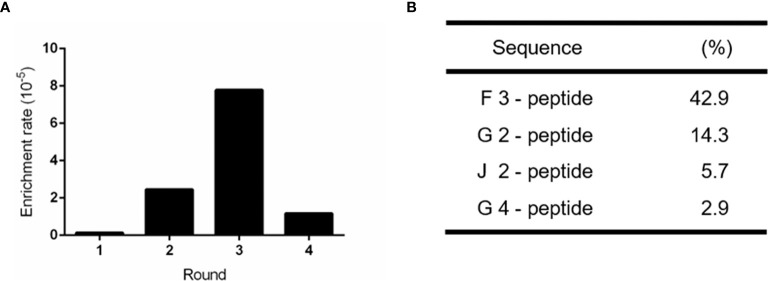
Enrichment of positive phage clones specifically binding to the recombinant human GPC-3 protein. **(A)** Bio-panning *via* a M13 phage-displayed C7C peptide library on the immobilized GPC-3 protein resulted in ~50-fold enrichment in the Round 3 of selection. **(B)** Highly repeated peptide sequences were obtained from the third round of bio-panned phages. 93 individual phage clones were randomly selected for sequencing after the third round of screening. The proportion of each positive peptide was shown.

To obtain a specific and high affinity GPC-3 binding peptide, four rounds of bio-panning were performed on the immobilized recombinant human GPC-3 protein (rhGPC-3) with the Ph.D.-C7C phage display library. With the increasing concentration of Tween-20 dissolved in PBST, the phage enrichment rate was improved gradually, suggesting that the GPC-3 binding phages were enriched effectively (**Figure 1A**). Subsequently, the fourth round of bio-panning was performed, and its enrichment rate showed a significant reduction compared to previous enrichment rates (**Table S1**). It indicated that the selection pressure reached its limitation through increasing concentrations of Tween-20 in PBST and three rounds of bio-panning were sufficient for the enrichment of GPC-3 binding phages. Phage clones recovered during Round 3 were randomly selected for DNA sequencing. The foreign cyclic peptide displayed on M13 phage was deduced from the DNA sequences based on triple-code theory. Analysis of the DNA sequencing data revealed that one cyclic peptide containing 7 amino acids restricted in two cystines was present at a high frequency, 42.9% among 93 individuals (**Figure 1B**), indicating its potential high affinity to rhGPC-3 protein for further research.

### Peptide Characterization

**Figure 2:**
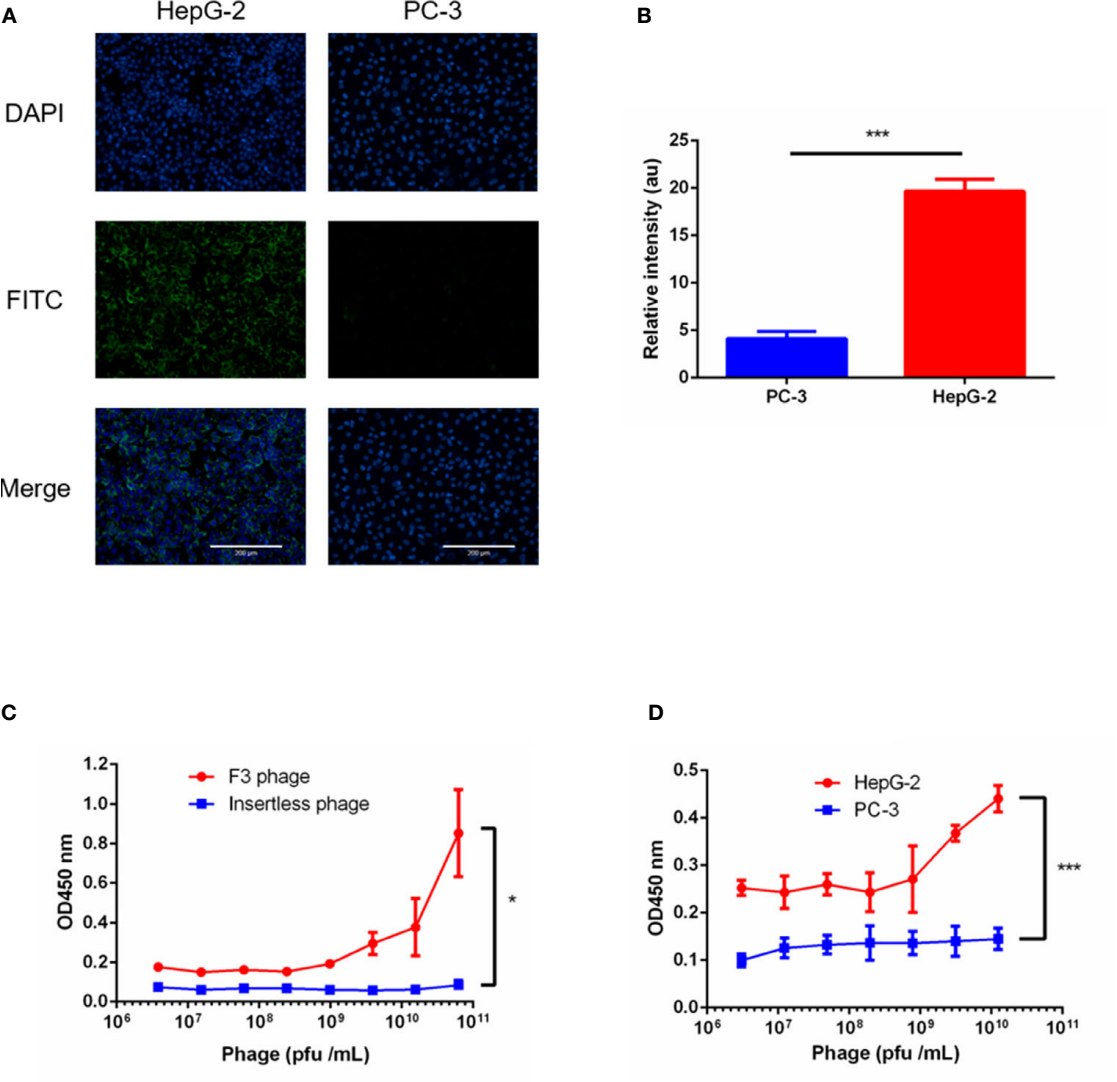
ELISA tests of phage clone F3 binding to GPC-3. **(A)** Immunofluorescence staining of GPC-3 expressed in the HepG-2 and PC-3 cell lines. Strong fluorescence was observed in HepG-2 cells, while barely signals were detected in PC3 cells as visualized by the anit-GPC-3 primary antibody and FITC-conjugated secondary antibody (green). Nuclei were stained with DAPI (blue). Scale bars: 200 µm. **(B)** Fluorescence signal of FITC in the PC-3 and HepG-2 cells was analyzed by the Image J software (n = 5 per group, p = 0.0002, ***p < 0.001, two-tailed Student’s t-test). **(C)** The ELISA test performed between phage clones and the recombinant human GPC-3 protein. The F3 phage showed high affinity to the GPC-3 protein. No concentration dependent binding between the insertless phage and the GPC3 protein was observed (n = 4 per group, p = 0.0255, *p < 0.05, two-tailed Student’s t-test). **(D)** The ELISA test performed between the F3 phage and cell lines. Stronger binding of the F3 phage was found in HepG-2 panels instead of PC-3 panels (n = 4 per group, p = 0.0001, ***p < 0.001, two-tailed Student’s t-test). All data represent the means ± standard error. P-value was calculated by two-tailed Student’s t-test.

Since we acquired a potential high affinity peptide, its homologous phage clone called Clone F3 was amplified and purified. The structure of the F3 peptide displayed as nine amino acids on the phage clone F3 was a cyclic peptide which contained 7 amino acids between two cystines forming a typical structure of a disulfide bond. To verify the binding affinity and specificity between the F3 peptide and GPC-3, the insertless (Int) M13 phage clone without displaying any foreign peptides were amplified and served as a control. Firstly, the expression of GPC-3 in HepG-2 and PC-3 cells was confirmed by immunofluorescence, and as shown in **Figures 2A** and **B**, higher expression of the GPC-3 protein was observed in HepG-2 cells while little GPC-3 protein in PC-3 cells was detected. Then, the HepG-2 and PC-3 cells were individually immobilized in the panel for the cell ELISA. As shown in **Figure 2D**, the phage clone F3 was able to bind HepG-2 cells instead of PC-3 cells in the ELISA test. Next, we performed another ELISA test between phage clones and the rhGPC-3 protein. With the lifting levels of the phage titer, Clone F3 was capable of strongly binding to the immobilized rhGPC-3 protein as present in **Figure 2C**. Meanwhile, Clone Int could barely bind to the same target protein. The binding affinity of F3 was 5.02 × 10^−5^ M (KD = 5.02 × 10**^-^** ^5^ M) obtained in the ELISA test against the rhGPC-3 protein. Above all, Clone F3 showed great affinity to GPC-3 due to its corresponding phage-displayed cyclic peptide.

### Validation of F3 Peptide Binding to Cell Lines *In Vitro*

**Figure 3:**
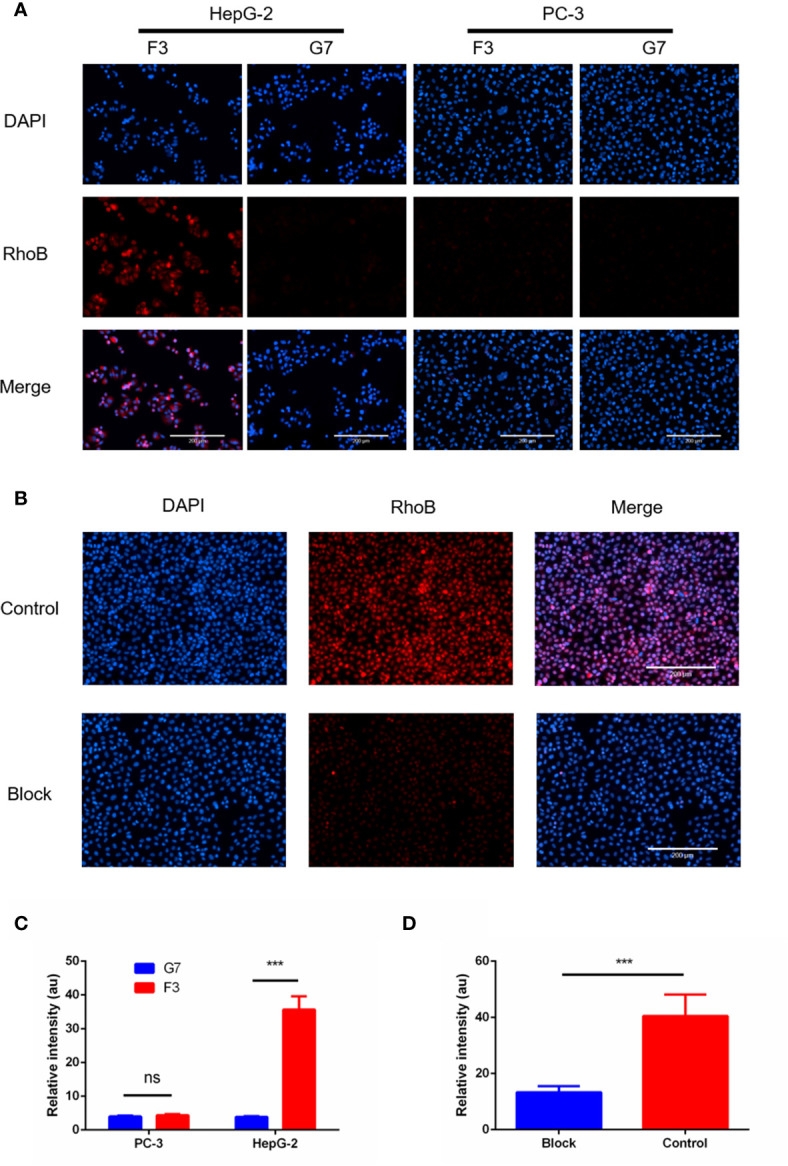
RhoB-F3 peptide targeting of GPC-3 (+) cell lines *in vitro*. **(A)** Fluorescent cell staining in the HepG-2 and PC-3 cell lines using the RhoB-conjugated F3 peptide. Specific binding of RhoB-F3 to HepG-2 cells could be observed as red fluorescence. Nuclei were stained with DAPI (blue). Scale bars: 200 µm. **(B)** The RhoB-F3 binding to HepG-2 cells could be blocked by the pre-incubated anti-GPC-3 primary antibody compared to the control group without being blocked. Nuclei were stained with DAPI (blue). Scale bars: 200 µm. **(C)** Quantification of RhoB fluorescence using the Image J in cell binding assay. F3 and G7 showed similar fluorescence intensity in PC-3 cells (n = 5, p = 0.0767, ns, not statistically significant, two-tailed Student’s t-test). The RhoB-F3 binding to the HepG-2 cells was much stronger than RhoB-G7 (n = 5 per group, p = 0.0001, ***p < 0.001, two-tailed Student’s t-test). **(D)** Quantification of RhoB fluorescence using Image J in the cell blocking assay. The binding activity between RhoB-F3 and HepG-2 cells could be effectively reduced by unlabeled F3 peptide (n = 5 per group, p = 0.0008, ***p < 0.001, two-tailed Student’s t-test). All data represent the means ± standard error. P-value was calculated by two-tailed Student’s t-test.

To confirm the binding capability of the F3 peptide to hepatoma cells, we synthesized F3 and C-G7-C peptide labeled with Rhodamine B (RhoB-F3 and RhoB-G7) for cell fluorescence staining. HepG-2 and PC-3 cells were incubated with RhoB-labeled peptides respectively at 37°C for 2 h. After washing off unbound peptides, the fluorescent signal of RhoB-labeled peptides binding to cells was detected and analyzed. As shown in **Figure 3A**, strong fluorescence was observed in HepG-2 cells incubated with RhoB-F3 while little fluorescence could be detected in PC-3 cells after incubation, indicating that the binding of F3 peptide to HepG-2 may depend on GPC-3 expression. The fluorescence intensity of RhoB-F3 attached to HepG-2 and PC-3 cells was significantly different as shown in **Figure 3C**. To further confirm the specificity of F3 peptide binding to GPC-3, a cell blocking assay was performed. The HepG-2 cells were previously incubated with an anti-GPC-3 primary antibody to block potential binding sites in the GPC-3 protein. After that, the RhoB-F3 peptides were added into the panel to compete with the primary antibody. The images of fluorescence detection in **Figure 3B** showed that the binding activity between RhoB-F3 peptides and HepG-2 cells could be effectively blocked by pre-incubation with the GPC-3 antibody (**Figure 3D**), suggesting that the specific binding target of F3 peptide is GPC-3.

### F3 Peptide Targeting to Hepatocellular Carcinoma *In Vivo*

**Figure 4:**
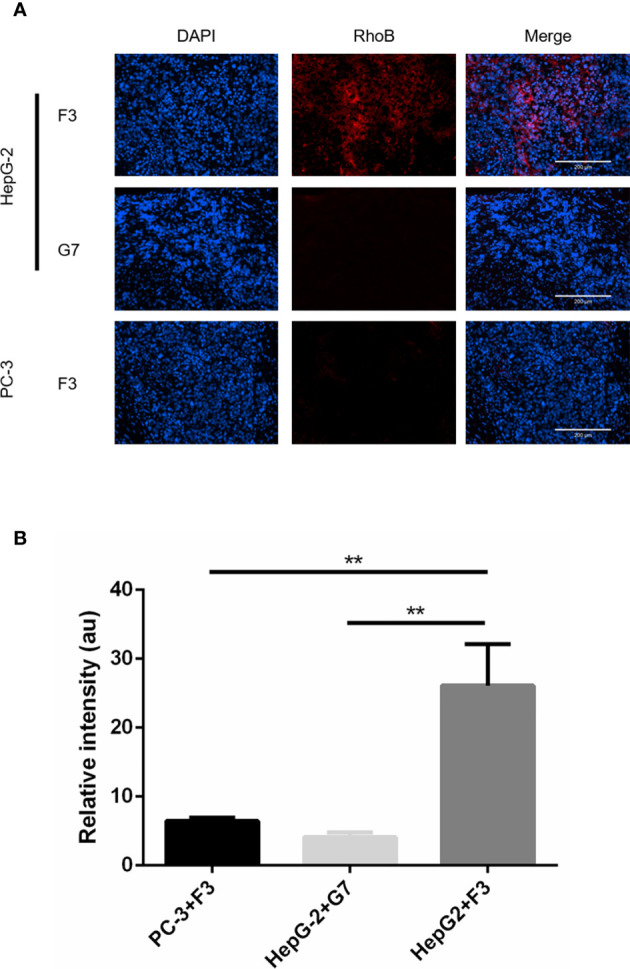
*In vivo* tumor targeting of RhoB-F3 peptide. **(A)** Fluorescent staining for subcutaneous tumor cryosections after 1 h intravenous injection of RhoB labeling peptides (red). Accumulation of RhoB-F3 (red) in HepG-2 tumors was much stronger than that in PC-3 tumors. Nuclei were stained with DAPI (blue). Scale bars: 200 µm. **(B)** Analysis of RhoB fluorescence intensity using Image J software. Accumulation of RhoB-F3 in PC-3 tumors and RhoB-G7 in HepG-2 tumors relative to RhoB-F3 in HepG-2 tumors (n = 5 per group, HepG-2+F3 to PC-3+F3, p = 0.0018, **p < 0.01, two-tailed Student’s t-test) (n = 5 per group, HepG2+F3 to HepG-2+G7, p = 0.0011, **p < 0.01, two-tailed Student’s t-test). All data represent the means ± standard error. P-value was calculated by two-tailed Student’s t-test.

To investigate the bio-distribution of F3 peptide, xenograft mouse models were established with the HepG-2 or PC-3 cells. After injection of RhoB-labeled F3 or G7 peptides through tail veins for 1 h, the tumor-bearing mice were sacrificed and major organs along with tumors were collected to study the distribution of the peptides *in vivo*. After the cryosections being made, a robust microscopic signal of RhoB-F3 was detected in HepG-2 tumors (**Figure 4A**), while minor fluorescent signal could be observed in other major organs, including the brain, lung, heart, digestive tracts, kidneys and bladder (**Figure S1**). Contrast to the strong fluorescent signal in HepG-2 tumors, significantly weaker signals of RhoB-F3 in the PC-3 tumors could be detected in tumor cryosections (**Figure 4B**). Meanwhile, as a control group, the RhoB-G7 peptide demonstrated almost no binding to the HepG-2 tumors (**Figure 4A**). In addition, the bio-safety of F3 peptide was preliminarily assessed as poisonless according to HE staining of major organs (**Figure S2**). Taken together, these results demonstrated that F3 peptide showed targeting accumulation in GPC-3 positive tumor tissues *in vivo*.

### Micro-PET/CT Imaging With ^68^Ga-DOTA-F3 in HCC Mouse Models

**Figure 5:**
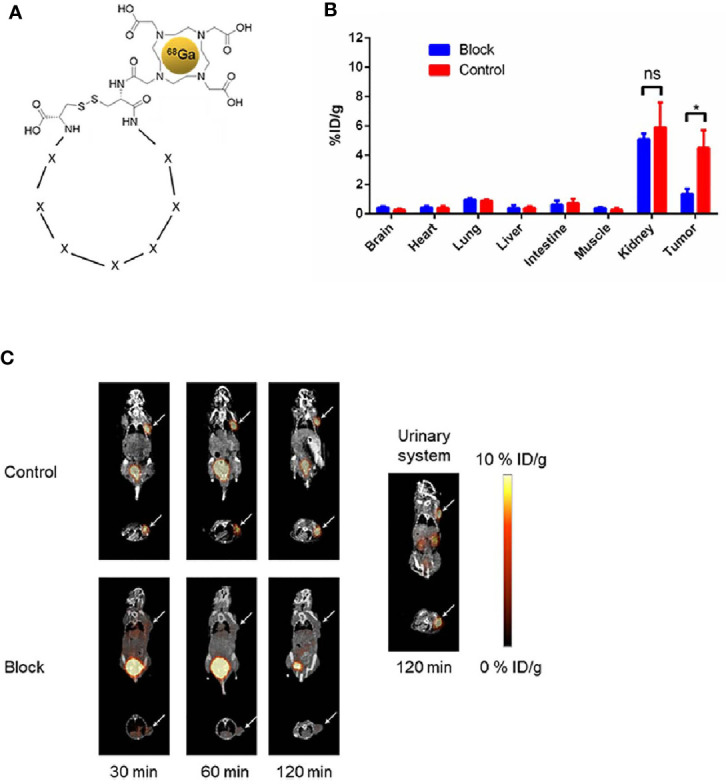
Micro-PET/CT imaging of ^68^Ga-DOTA-F3 targeting HepG-2 tumor *in vivo*. **(A)** Chemical structure of ^68^Ga-DOTA-F3. F3 peptide containing 7 amino acids (represent as X) restricted in two cystine was modified with DOTA at the N-terminus. **(B)** Quantification of ROIs in HepG-2 xenografted mice after injection for 60 min presented as %ID/g. The uptake of ^68^Ga-DOTA-F3 could be sufficiently blocked by pre-injection of DOTA-F3 without radiation (n= 3 per group, p = 0.0121 in tumors of two groups, *p < 0.05, two-tailed Student’s t-test) (n= 3 per group, p = 0.4708 in kidneys of two groups, ns, not statistically significant, two-tailed Student’s t-test). All data represent the means ± standard error. P-value was calculated by two-tailed Student’s t-test. **(C)** The whole-body coronal micro-PET/CT images of HepG-2 tumor bearing mice at 30, 60 and 120 min pi. Metabolism of radiotracer by urinary system could be apparently observed at 120 min pi. Tumors are indicated by arrows.

To construct the ^68^Ga-DOTA-F3 as a radiotracer, the DOTA-F3 peptide was synthesized and labeled with radioactive ^68^Ga according to the structure shown in **Figure 5A**. The radioactivity of ^68^Ga-labeled peptide was steady at a concentration of 25.90 MBq/mg, and the labeling efficiency (RCY%) for ^68^Ga-DOTA-F3 was around 80% (**Table S2**) without the need for further HPLC (High Performance Liquid Chromatography) purification. Mice bearing HepG-2 tumors were intravenously injected with 7.40 MBq of ^68^Ga-DOTA-F3 *via* the tail vein. The Micro-PET/CT was performed after circulation of ^68^Ga-DOTA-F3 for 30 min, 60 min and 120min respectively. As the PET/CT images shown in **Figure 5C**, ^68^Ga-DOTA-F3 could accumulate in HepG-2 subcutaneous tumors with strong expression of GPC-3 during circulation. The highest uptake of tumors and the lowest uptake of background at the same time were observed at 60 min post injection, suggesting that it might be a appropriate timing for tumor imaging and diagnosis. Meanwhile, metabolic accumulation in the kidneys and the bladder indicated the major clearance of the radiotracer relying on the urinary system. After circulation for 60 min, radioactivity of accumulation in the tumor and major organs was quantified and analyzed by measuring the ROIs which encompassed the entire organ on coronal images of the control group. The HepG-2 tumor uptake of ^68^Ga-DOTA-F3 was calculated to be 4.50 ± 1.20%ID/g while the kidney uptake value was 5.90 ± 1.70%ID/g and the background level was considered to be 0.27 ± 0.14%ID/g (**Figure 5B**). To block the binding activity of radio-labeled F3 peptide to HepG-2 tumors, unlabeled F3 peptide was intravenously pre-injected allowing circulation for 60min before applying ^68^Ga-DOTA-F3. Consequently, a dramatic reduction of PET signal could be observed in the HepG-2 subcutaneous tumor (1.36 ± 0.35%ID/g) during circulation in the blocking assay (**Figures 5B, C** **)**, suggesting that the specific uptake of the HepG-2 tumor was dependent on F3 peptide.

### Detection of GPC-3 With F3 Peptide *Ex Vivo*

**Figure 6:**
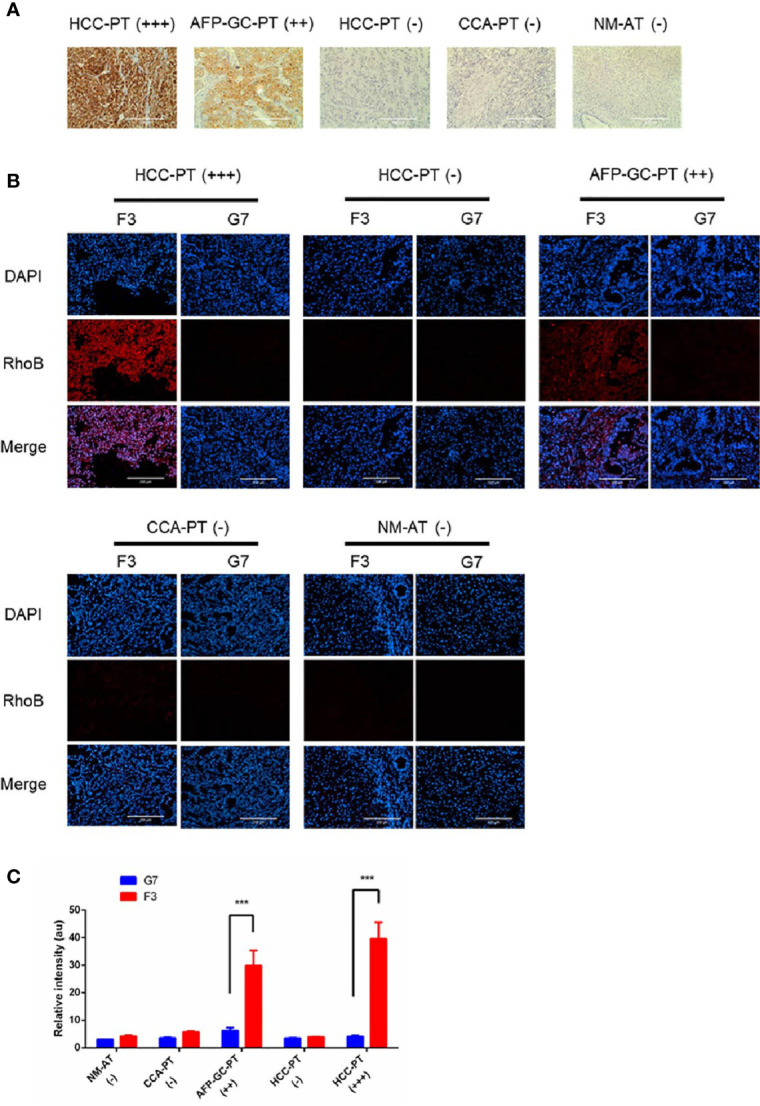
Detection of GPC-3 in clinical samples ex vivo with RhoB-F3 peptide. **(A)** Immunohistochemical staining of GPC-3 in human tissues with anti-GPC-3 antibody. Expression levels of GPC-3 were evaluated as (–), (++), and (+++). Scale bars: 200 µm. **(B)** Fluorescent staining with RhoB labeling peptides (red) in sections. Strong binding of RhoB-F3 to AFP-GC-PT (++) and HCC-PT (+++) was noticed. Nuclei were stained with DAPI (blue). Scale bars: 200 µm. **(C)** Quantification of RhoB fluorescence using Image J. F3 peptide showed great affinity to AFP-GC-PT and HCC-PT relative to G7 peptide (n = 5 per group, F3 to G7 in HCC-PT (+++), p =0.0002, ***p < 0.001, two-tailed Student’s t-test) (n = 5 per group, F3 to G7 in AFP-GC-PT, p =0.0001, ***p < 0.001, two-tailed Student’s t-test). All data represent the means ± standard error. P-value was calculated by two-tailed Student’s t-test.

To investigate the potential clinical application of the F3 peptide, we performed a fluorescence staining assay in various human tissue samples. Tumor tissues from HCC patients, CCA patients and AFP-GC patients were noted as HCC-PT, CCA-PT and AFP-GC-PT respectively, besides, the normal adult liver tissue was also named as NM-AT. First of all, the expression of GPC-3 protein was evaluated by IHC assays. As shown in **Figure 6A**, the GPC-3 protein was highly expressed in HCC-PT and AFP-GC-PT, but absent in CCA-PT and NM-AT. Next, the RhoB-F3 peptide was applied in fluorescence staining of cryosections. As demonstrated in **Figures 6B, C**, significant fluorescent signals of RhoB-F3 peptide were detected in the HCC-PT and APF-GC-PT, but absent in either CCA-PT or NM-AT. These results were consistent with corresponding levels of the GPC-3 protein expressed in various tissue samples, indicating a promising future of F3 peptide in clinical application. To further confirm the selective binding of Rho-F3 peptide, the RhoB-G7 peptide was also applied in fluorescence staining assays, which showed weak fluorescent signals close to background signals (**Figure 6B**). Based on the specificity and high affinity to GPC-3, the F3 peptide exhibits promising value in clinical application. To ensure the bio-safety of clinical application in the future, the selective binding to human normal organs, including the brain, heart, lung, digestive tract, spleen, bladder and artery, should be seriously considered in preclinical assessment. So we investigated the selective binding ability between the RhoB-P3 peptide and human normal tissues. As shown in **Figure S3**, no detectable signals of the RhoB-F3 could be observed in normal tissues, suggesting its weak affinity to human normal organs. In conclusion, the F3 peptide could bind to GPC-3 in human cancer tissues specifically and showed great potential for further translational research.

## Discussion

HCC, a common malignancy in the digestive system, presents a rapid increase in incidence and mortality, which is often diagnosed at an advanced stage associated with poor prognosis (18). A variety of therapeutic strategies, including radiotherapy, chemotherapy and surgery, are working to improve the treatment of HCC patients. Although the revolution of immunotherapy has come and partial patients have benefited from the immune checkpoint blockades, five-year survival rate of HCC is unfavorable, suggesting that there are still great challenges in HCC diagnosis and treatment (19, 20). Recent studies focusing on targeting GPC-3 in HCC showed inspiring results in precise imaging and targeting therapy, eliciting great attention to biological agents aimed at GPC-3 (21–23). Phage display system, a powerful platform for obtaining high affinity peptides to a target, has been explored in recent decades for clinical application in precise diagnosis and treatment of various diseases (24, 25). Hence, the investigation of functional peptides generated from the phage display platform has attracted concerns from researchers.

In this study, we identified a specific phage clone binding to GPC-3 and found that the F3 peptide exhibited high affinity and specificity to the GPC-3 protein. F3 peptide could successfully distinguish GPC-3 in different cell lines and human tumor samples. More importantly, the F3 peptide labeled with radioactive ^68^Ga could successfully detect GPC-3-positive HCC tumors *in vivo* and the PET imaging analysis revealed a high uptake of tumor compared to the minor background accumulation (4.50 ± 1.20 to 0.27 ± 0.14% ID/g). These results indicated that this tumor-targeting peptide might contribute to precise imaging and targeting therapy in HCC patients, such as peptide-drugs conjugation, targeting-nanoparticles and other application.

In the past decades, several monoclonal and bispecific antibodies against GPC-3 have been developed for HCC imaging and therapy (26, 27). One preclinical study has been carried out by Waaijer SJ using a ^89^Zr-labeled bispecific antibody against CD3 and GPC-3, and a PET imaging was performed after 24 h for circulation of the radiotracer (28). Due to the massive molecular weight of the antibody, the detection of GPC-3 in HCC had to be delayed for an extended circulation time of the tracer for its background clearance. Such antibody-based imaging strategies may have several intrinsic drawbacks to overcome, including prolonged half-life time and metabolic accumulation in the liver. Additionally, the antibody-based imaging cost extra days to ensure sufficient clearance of background radioactivity for precise tumor imaging. As a result, patients preparing for imaging may suffer from time consuming and inconvenience, thus making poor compliance of patients. In contrast, our peptide-based PET imaging showed quick background clearance within 2 h depending on the metabolism of the urinary system, and could target the GPC-3 protein in HCC tumors. When it comes to targeting peptide applied in PET imaging, another peptide named TJ12P2 targeting GPC-3 has been successfully developed and applied in the Micro-PET/CT (29). Although the TJ12P2 peptide could greatly accumulate in GPC-3 positive tumor tissues, there was still some moderate diffusion of residue probes in the abdominal cavity which might be considered as background signals. Compared with the TJ12P2 peptide, our F3 peptide seemed to prefer accumulation more in tumors and showed less diffusion in other cavities. More importantly, we did a bio-safety assessment in normal adult tissues and the binding of the F3 peptide could be barely observed indicating its potential clinical safety. Besides, biological macromolecules like antibodies usually fail to diffuse freely in solid tumors, resulting in incomplete penetration (30, 31). Hence, inspired by tumor-penetrating peptide (32, 33), cyclic tumor-targeting peptide should be considered to provide high affinity accompanied by the penetration ability due to its low molecular weight and annular conformation. The penetration ability of the F3 peptide will be explored in 3D tumor spheroids and further modification of the peptide will be our focus.

In our study, the F3 peptide showed great affinity to GPC-3 positive tumor cells *in vitro*, *ex vivo* and *in vivo*. Meanwhile, highly selective binding to tumor tissues enabled the F3 peptide promising clinical application, reducing off-target side effects in normal organs. In conclusion, we identified a tumor-targeting peptide specific for GPC-3 with high affinity and specificity. The F3 peptide targeting GPC-3 could distinguish GPC-3 positive tumors in different cancer types. The ^68^Ga-labeled PET imaging tracer targeting GPC-3, ^68^Ga-DOTA-F3, might hold promise for peptide-based PET imaging in patients with HCC.

## Data Availability Statement

The original contributions presented in the study are included in the article/**Supplementary Material**. Further inquiries can be directed to the corresponding author.

## Ethics Statement

The studies involving human participants were reviewed and approved by Nanjing Drum Tower Hospital Ethics Committee. The patients/participants provided their written informed consent to participate in this study. The animal study was reviewed and approved by Institutional Animal Care and Use Committee of Nanjing Drum Tower Hospital.

## Author Contributions

Conceptualization, JW and BL. Methodology, JY, XY, and XC. Investigation, JY, FL, FC, and ND. Writing—review & editing, JY, JS, and BL. Funding acquisition, JW and BL. Supervision, LY, FM, JW, and BL. All authors contributed to the article and approved the submitted version.

## Funding

This work was funded by grants from the National Natural Science Foundation of China (no. 81930080), Program of Jiangsu Provincial Key Medical Center (no. YXZXB2016002), National Natural Science Foundation of China (no. 82072926), and National Natural Science Foundation of China (no. 81902911). The funding sources had no role in the study design, data collection, data analysis, data interpretation, or writing of the report.

## Conflict of Interest

The authors declare that the research was conducted in the absence of any commercial or financial relationships that could be construed as a potential conflict of interest.
