# Supplementary material for: Identification of a Glypican-3 Binding Peptide From a Phage-Displayed Peptide Library for PET Imaging of Hepatocellular Carcinoma
Source: Front Oncol. 2021 Jun 4;11:679336. doi: 10.3389/fonc.2021.679336 (PMC8212053; doi:10.3389/fonc.2021.679336)
Supplement: Supplementary file 1 [file DataSheet_1.pdf]

Table S1 Enrichment of phages from each round biopanning

Round	Input Phage[a] (pfu)	Output Phage[b] (pfu)	Enrichment rate[c]
1	2.0×10^{11}	8.8×10^{4}	4.4×10^{-7}
2	1.0×10^{12}	1.2×10^{6}	1.2×10^{-6}
3	9.0×10^{11}	5.2×10^{6}	5.8×10^{-6}
4	3.8×10^{11}	5.3×10^{5}	1.4×10^{-6}

[a] Number of phages prepared at the start of a biopanning round.
[b] Number of phages recovered at the end of a biopanning round.
[c] The enrichment rate was calculated by the output/input ratio of phages recovered after each round of biopannning.

Table S2 Labeling efficiency for ^{68}Ga-DOTA-F3

Radioactivity	Input[1] (MBq)	Waste[2] (MBq)	C18 Column[3] (MBq)	Product[4] (MBq)	Labeling efficiency[5]
Test 1	93.24	10.36	9.25	85.84	92.1%
Test 2	157.47	11.10	12.95	128.02	81.3%
Test 3	168.27	7.40	17.76	132.09	78.5%

[1] Original radioactive $^{68}GaCl_3$ for reaction
[2] Waste liquid abandoned after purification
[3] Residue mixture attached on C18 plus light cartridge
[4] Collection of purified radiotracer
[5] Labeling efficiency (Radio-chemical yield %) = Product radioactivity / Input radioactivity.

Figure S1

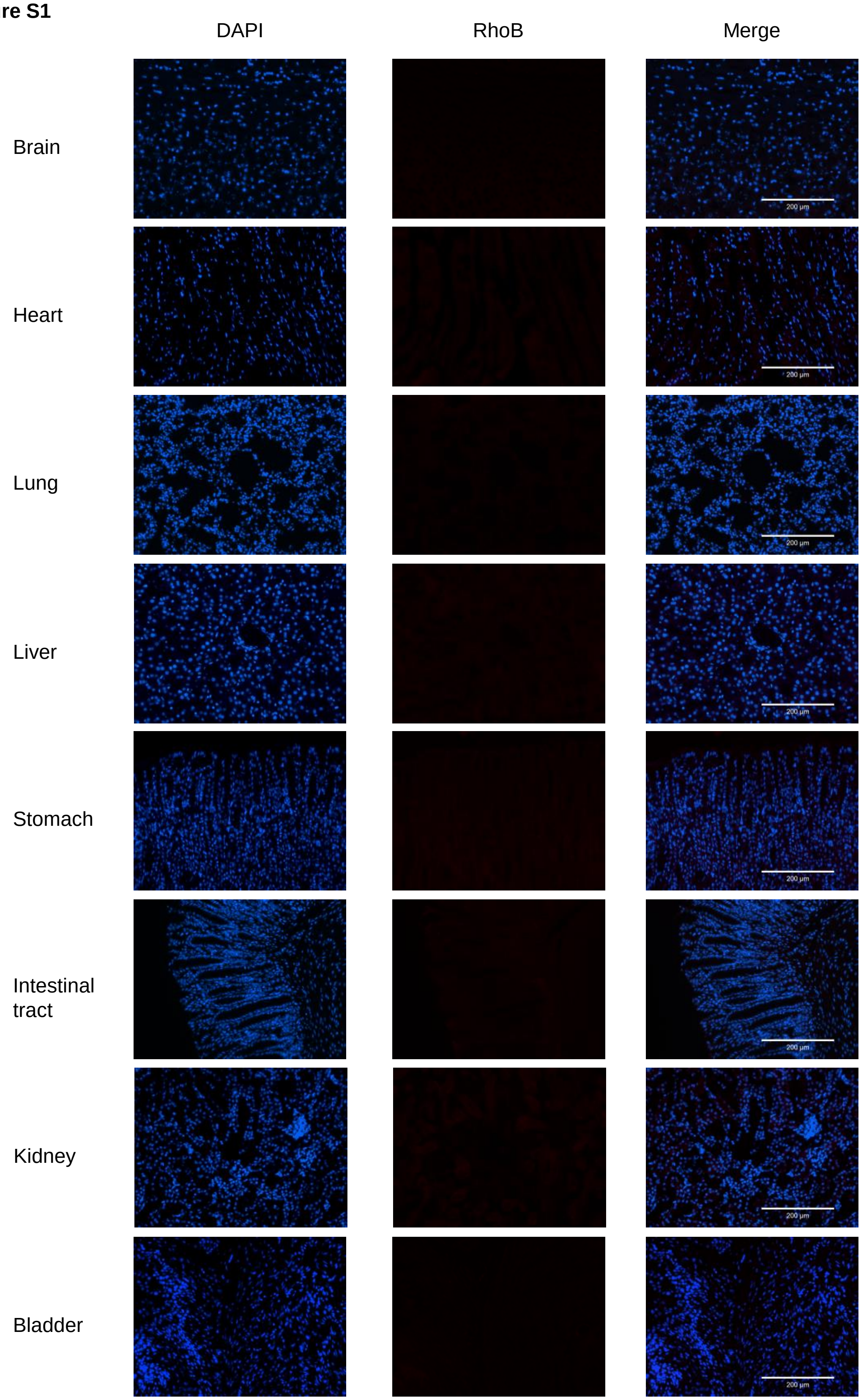
DAPI
RhoB
Merge
Brain
Heart
Lung
Liver
Stomach
Intestinal tract
Kidney
Bladder

Figure S2

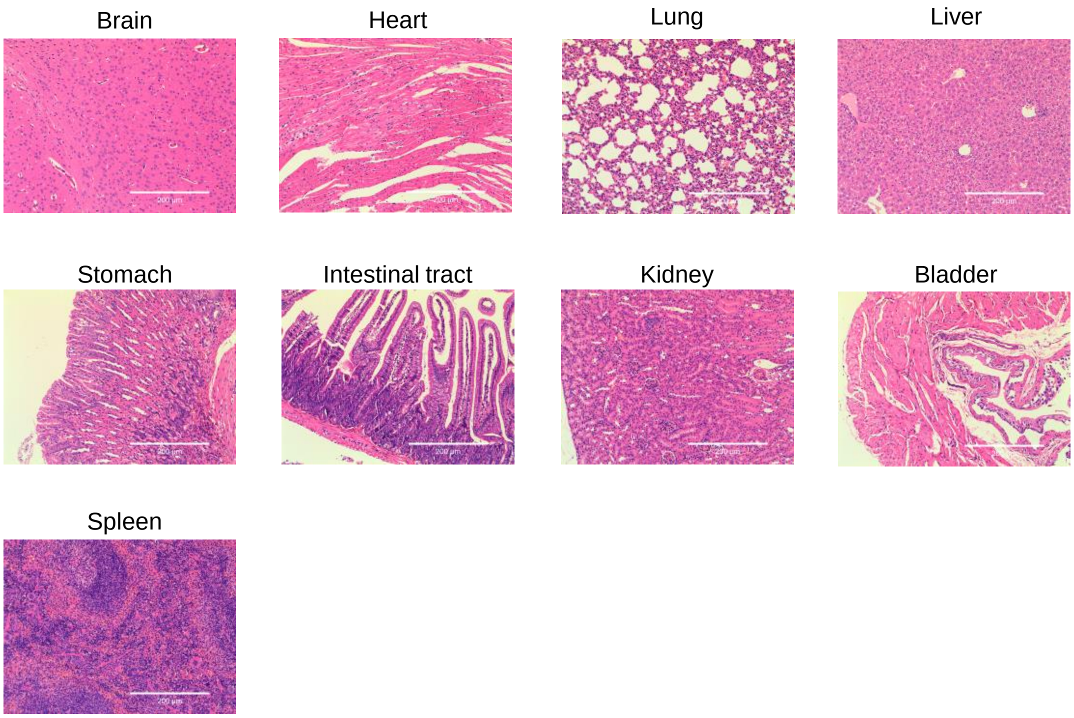
Brain
Heart
Lung
Liver
Stomach
Intestinal tract
Kidney
Bladder
Spleen

Figure S3

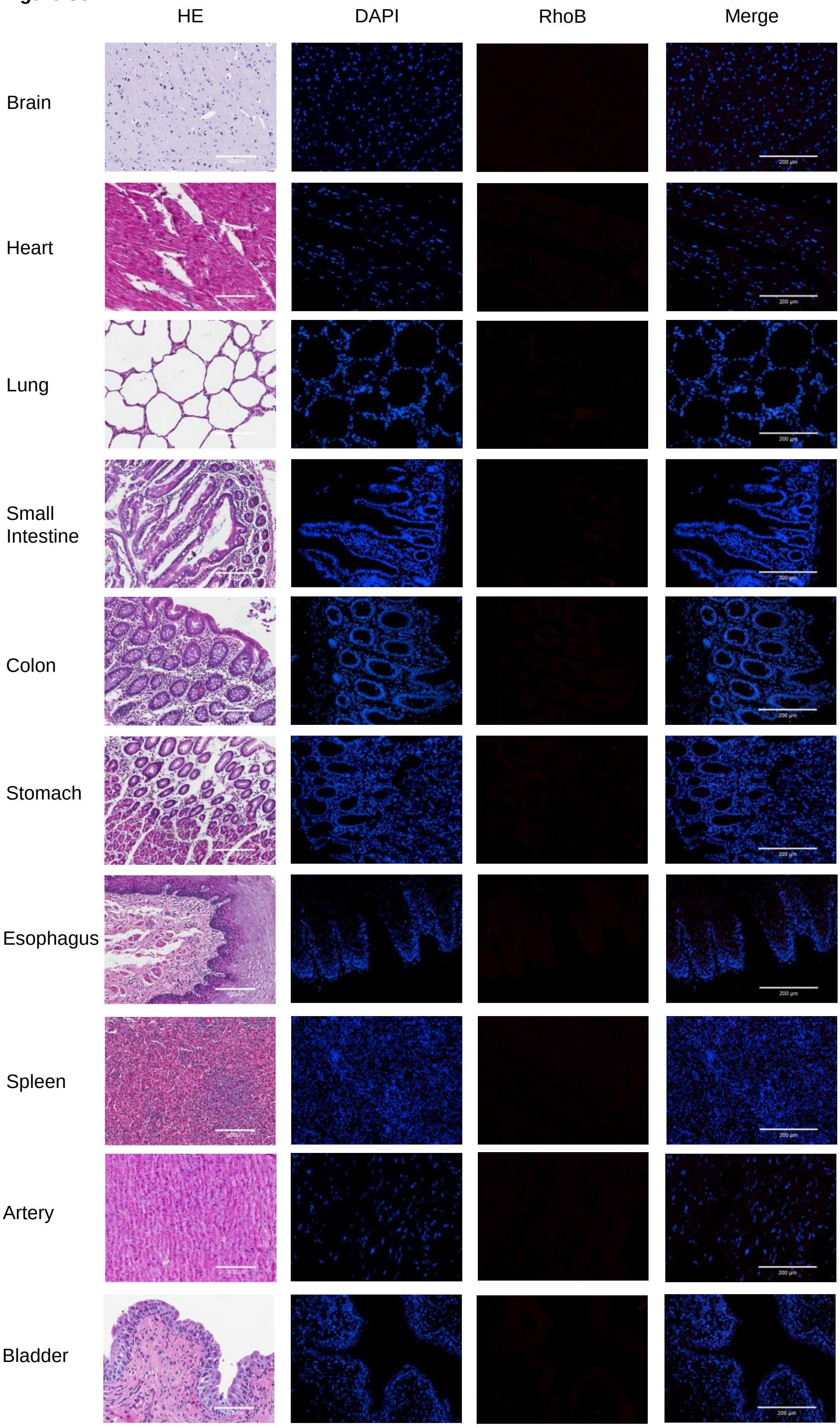

HE
DAPI
RhoB
Merge
Brain
Heart
Lung
Small Intestine
Colon
Stomach
Esophagus
Spleen
Artery
Bladder

Supplemental Material Figure Legends

Figure S1. Biodistribution of RhoB-F3 in HepG-2 tumor bearing mice. Fluorescence of RhoB presented as red color. Nuclei were stained with DAPI (blue). Scale bars: 200 µm.

Figure S2. HE staining of several organs from HepG-2 tumor bearing mice injected with RhoB-F3. Scale bars: 200 µm.

Figure S3. HE staining of human normal tissue samples. Scale bars: 400 µm. Fluorescence staining with RhoB-F3 (red) in human normal tissue sections. Nuclei were stained with DAPI (blue). Scale bars: 200 µm.
